# Association of chronic hepatitis B infection with hepatic steatosis and injury in nonalcoholic fatty liver disease children

**DOI:** 10.1186/s12876-023-03103-9

**Published:** 2024-01-02

**Authors:** Lu Wang, Chang Lu, Yuncong Zhang, Qingsheng Liang, Jie Zhang

**Affiliations:** 1 Department of Laboratory Medicine, Peking University International Hospital, Zhongguancun Life and Science Street NO.1, 102206 Beijing, People’s Republic of China; 2 Center of Non-Infectious Liver Disease, the 5Th Medical Centre, Chinese PLA General Hospital, Beijing, 100039 People’s Republic of China

**Keywords:** Nonalcoholic fatty liver disease (NAFLD), Chronic hepatitis B infection (CBI), Pediatric population

## Abstract

**Background:**

The influence of chronic hepatitis B infection (CBI) on hepatic steatosis, necroinflammation, and fibrosis in nonalcoholic fatty liver disease (NAFLD) population was unclear. We aimed to investigate the effect of CBI on hepatic steatosis and assess the association between NAFLD co-existed CBI and hepatic injury in NAFLD pediatric population.

**Methods:**

Consecutive hospitalized children with biopsy-proven NAFLD with or without CBI were included. Hepatic steatosis, necroinflammation and fibrosis were evaluated by NASH CRN system and/or METAVIR scoring system, appropriately. Using multivariate logistic analysis, we identified variables associated with hepatic steatosis and liver injury.

**Results:**

Of 223 biopsy-proven NAFLD children, 161 were NAFLD without CBI, and 62 were NAFLD co-existed CBI. Grouped by mild, moderate and severe hepatic steatosis, there was an inverse association between CBI and the severity of hepatic steatosis [odd ratio (OR) 0.037, 95% confidence interval (CI) 0.014–0.098]. In addition, we explored the relationship between CBI and hepatic necroinflammation and fibrosis in NAFLD children. Hepatic necroinflammation and fibrosis, respectively, were divided into two groups according to severity. And CBI was positively associated with hepatic necroinflammation (OR 6.125, 95%CI 1.958–19.158). However, there was no statistically independent association between CBI and significant hepatic fibrosis.

**Conclusions:**

CBI was inverse associated with the grade of steatosis and positively associated with severe hepatic necroinflammation, and does not appear to affect significant hepatic fibrosis in pediatric NAFLD children.

**Supplementary Information:**

The online version contains supplementary material available at 10.1186/s12876-023-03103-9.

## Background

Nonalcoholic fatty liver disease (NAFLD)is the most common metabolic liver disease worldwide with a prevalence of 25.24% globally [1]. Given that NAFLD is strongly associated with obesity, the disease incidence in children has shown an upward trend consistent with economic growth and lifestyle changes. The clinic-histopathological spectrum of NAFLD covers nonalcoholic fatty liver (NAFL), non-alcoholic steatohepatitis (NASH), and cirrhosis. All the three diseases could potentially lead to NAFLD-related hepatocellular carcinoma (HCC) [2]. Liver biopsy is the gold standard for accurate diagnosis and staging of NAFLD, especially for children without clear predisposing factors of metabolic syndrome, and for those with suspected hepatic fibrosis. Compared to NAFLD in adults, childhood NAFLD presents several different features including more abundant or accentuated hepatocyte steatosis in zone 1, uncommon ballooning, and initial fibrosis and necroinflammation in portal tracts [3]. Furthermore, NAFLD in children has been reported with a worse prognosis as compared to that in adults, which might correlate to increasing morbidity and mortality in adulthood [2, 4].

Meanwhile, Hepatitis B virus (HBV) infection is the most common reason for chronic liver disease, affecting approximately 257 million infections worldwide [5]. Although the implementation of HBV immunization strategies and the efficient blockage of mother-to-child transmission (MTCT) has significantly reduced the incidence of HBV infection, a considerable number of children are still affected by chronic HBV infection (CBI) due to the huge population of HBV infection and the certain failure rate of blockage of MTCT in China [6, 7]. Most chronic hepatitis B infection (CBI) children are in immune tolerance phase accompanied by minor histological changes [8]. Long-standing HBV has long been recognized as a significant risk factor for hepatic necroinflammation and its further progression to hepatic fibrosis, cirrhosis and HCC.

Both NAFLD and CBI are conditions generally associated with hepatic necroinflammation, hepatic fibrosis, cirrhosis and liver cancer. Emerging epidemiological evidence has demonstrated that CBI is inversely associated with hepatic steatosis [9, 10]. Consistently, our previous study also found a reciprocally inverse association between NAFLD and CBI existence in pediatric population [11]. Steatosis or severe steatosis has been reported to be positively associated with severity of fibrosis in chronic hepatitis B (CHB) [12, 13]. However, recent studies have also reported opposite conclusionsthat steatosis is not associated with hepatic fibrosis in CHB population [14–16]. As described above, liver biopsy plays as the gold standard for the diagnosis of hepatic steatosis, necroinflammation and fibrosis assessment, which could be leveraged to further investigate the effect of CBI on NAFLD-related fibrosis and cirrhosis. Furthermore, pathophysiological and virologic characteristics show significant differences between childhood and adulthood NAFLD. A higher frequency of poor outcomes in pediatric NAFLD has been observed in comparison to adult populatio. Thus, it is worth exploring if NAFLD with overlapping CBI could accelerate or suspend the progression of liver disease in NAFLD population, especially in pediatric patients. In this study, we the effect of CBI on the severity of hepatic steatosis, hepatic necroinflammation, hepatic fibrosis and cirrhosis in NAFLD pediatric population in light of histopathological information through the application of liver biopsy.

## Methods

### Patients selection

NAFLD children (0–18 years) with or without CBI by percutaneous liver biopsies were retrospectively and consecutively included from January 2010 to March 2022 at the 5th Medical Center, Chinese PLA General Hospital. Meanwhile, NAFLD co-existed CBI children were treatment naïve individuals and excluded with: (1) malignant hepatic tumor, (2) acute hepatitis B, (3) anti-HBV treatment, (4) suspected or confirmed hepatolenticular degeneration, (5) co-infection with other viruses, (6) other hepatic disease or severe systemic disease.

To ensure the clinical and laboratory data is directly matched with the histological specimens, only children with clinical and laboratory data collected within two days of the biopsy and did not receive any treatment were included. Liver biopsy with more than 12 mm length were formalin-fixed and paraffin-embedded and subsequently stained with hematoxylin–eosin safran and Masson’s trichrome.

The diagnosis of NAFLD was based on the NAFLD Practice Guidance from the American Association for the Study of Liver Diseases (AASLD) [1]. Chronic HBV infection (CBI) was defined by positive serum hepatitis B surface antigen (HBsAg) for 6 months or more according to AASLD 2018 Hepatitis B Guidance [17]. Metabolic syndrome was diagnosed in accordance with IDF consensus report [18].

### Histological assessment

Histological liver samples were assessed separately by two experienced pathologists.

NAFLD children were histological evaluated according to NASH CRN system. Individuals with NAFLD co-existed CBI were evaluated by METAVIR scoring system for hepatic necroinflammation and fibrosis and by NASH CRN system for steatosis grading. Briefly, Hepatic steatosis was graded as: mild = 5–33% steatosis, moderate = 34%-66% steatosis, severe =  > 67% steatosis. Hepatic fibrosis was staged: F0 = no fibrosis, F1 = portal fibrosis without septa, F2 = portal fibrosis with rare septa, F3 = numerous septa without cirrhosis, and F4 = cirrhosis. Hepatic necroinflammation was graded: A0 = no activity, A1 = mild activity, A2 = moderate activity, A3 = severe activity [19]. All children or their parents/ guardians have written informed consent before liver biopsy.

### Statistical analysis

Continuously demographic and clinical variables were presented as median and interquartile range and were compared using nonparametric Mann–Whitney *U* test. Categorical variables were expressed as percentages and were judged the differences between the groups using Chi-square test or Fisher’s exact test as appropriate. Multivariate logistic regression was carried out to establish independent factor of hepatic steatosis, hepatic necroinflammation and fibrosis, in which factors with *P* < 0.05 in the univariate model were entered into enter multivariate model, besides, age, gender, BMI z-score, ALT and GGT that were entered into multivariate model. Variance inflation factor testing was used for the detection of multicollinearity and the value was no more than 10. A two-tailed *P* value < 0.05 was considered as statistically significant. All statistical data were analyzed using SPSS software for Windows version 26.0 (SPSS Inc., Chicago, IL, USA).

### Ethical statement

The investigation was performed in accordance with the guidelines of the Declaration of Helsinki and has been approved by the Ethics Review Team of the Fifth Medical Center of the Chinese PLA General Hospital (approval number: KY- 2022–1-5–1).

## Results

### Characteristics of children

Our study included 223 pediatric individuals diagnosed with nonalcoholic fatty liver disease, of which 161 children were diagnosed NAFLD without chronic hepatitis B infection (CBI) and 62 were co-existing NAFLD and CBI. In the 161 NAFLD without CBI children, 12 (7.5%) were mild hepatic steatosis, 40 (24.8%) were moderate steatosis and 109 (67.7%) were severe steatosis. While, in co-existing CBI group, 46 (73.2%) acquired mild steatosis, 11 (17.7%) were moderate and only 5 (8.1%) were severe steatosis. (*P* < 0.001) 98/161 (60.9%) mild hepatic necroinflammation (A0-1) were found in NAFLD without CBI children and 28/62 (48.4%) were in NAFLD with CBI children (*P* = 0.034). However, there were no significant differences in hepatic fibrosis between NAFLD with and without CBI group (*P* = 0.738). Other clinical characteristics of NAFLD with and without CBI individuals were summarized in Table 1.

**Table 1:** Characteristics of NAFLD pediatric population

Variable	NAFLD without CBI (*n* = 161)	NAFLD with CBI (*n* = 62)	*P*-value*
	**M (quartile)**	**M (quartile)**	
Age (years)	12.0 (10.0–14)	11.0 (6.8–15.3)	0.606
Gender (M/F)	141/20	54/8	0.923
BMI z-score	-0.05 (-0.91–0.47)	0.18 (-0.42–0.82)	0.04
Platelet (10^9/L)	284 (244–334)	248 (208–286)	< 0.001
Albumin (g/L)	45.0 (43.0–47.0)	42.5 (40.0–47.0)	0.018
Globulin	27 (24–29)	24 (27–29)	0.321
Prealbumin (mg/L)	232 (207–263)	151 (137–185)	< 0.001
ALT (U/L)	143 (85–237)	87.0 (51–124)	< 0.001
AST (U/L)	79 (49–128)	68 (46–87)	0.120
GGT(U/L)	60 (42–91)	25 (21–45)	< 0.001
TBA (umol/L)	6 (3–8)	7 (5–13)	0.002
Creatinine (umol/L)	55 (47–63)	49 (44–61)	0.050
Cholinesterase (U/L)	9822 (8579–10,609)	7359 (6710–8925)	< 0.001
Urid acid (umol/L)	382 (310–443)	291 (271–369)	< 0.001
Fibrinogen (g/L)	3.10 (2.57–3.53)	2.74 (2.22–3.28)	0.020
PT (s)	10.9 (10.4–11.4)	11.4 (10.9–12.1)	< 0.001
TC (mmol/L)	4.36 (3.85–4.97)	3.7 (3.4–4.3)	< 0.001
TG (mmol/L)	1.47 (1.15–1.97)	0.90 (0.78–1.28)	< 0.001
HDL-C (mmol/L)	1.10 (0.99–1.31)	1.20 (1.03–1.29)	0.189
LDL-C (mmol/L)	2.96 (2.55–3.45)	2.31 (2.06–2.84)	< 0.001
ApoA1 (mmol/L)	1.22 (1.12–1.37)	1.28 (1.15–1.38)	0.191
ApoB (mmol/L)	0.81 (0.67–0.97)	0.61 (0.53–0.72)	< 0.001
Lp (a) (mmol/L)	46 (29–80)	62 (29–76)	0.291
Metabolic syndrome (n, %)	27 (18.9%)	1 (1.6%)	0.001
**Hepatic steatosis**			< 0.001
Mild (n, %)	12 (7.5%)	46 (73.2%)	
Moderate (n, %)	40 (24.8%)	11 (17.7%)	
Severe (n, %)	109 (67.7%)	5 (8.1%)	
**Stage of Fibrosis**			0.928
S0-1 (n, %)	79 (49.1%)	30 (48.4%)	
S2-4 (n, %)	82 (50.9%)	32 (51.6%)	
**Grade of Necro**			0.034
A0-1 (n, %)	98 (60.9%)	28 (45.2%)	
A2-3 (n, %)	63 (39.1%)	34 (54.8%)	
HBsAg(COI)	-	2162 (1108–5256)	
HBeAg(COI)	-	410 (6.4–1026)	
HBV DNA (copy/ml)	-	8.79 (0.84–23.53) × 10^7^	
HBV Genotype			
B	-	12	
C	-	34	

*Abbreviation*: *NAFLD* Nonalcoholic fatty liver disease, *CBI* Chronic hepatitis B infection, *BMI* Body mass index, *ALT* Alanine aminotransferase, *AST* Aspartate aminotransferase, *GGT* Glutamyl transferases, *TBA* total bile acid, *TC* Total cholesterol, *TG* Triglyceride, *HDL-C* High density lipoprotein cholesterol, *LDL-C* Low density lipoprotein cholesterol, *ApoA1* Apolipoprotein A1, *ApoB* Apolipoprotein B, *Lp (a)* Lipoprotein (a), *PT* Prothrombin time, *Grade of Necro.* Grade of necroinflammation, *HBV* Hepatitis B virus, *HBsAg* Hepatitis B surface antigen, *HBeAg* Hepatitis B e antigen

“*” means the *p* value between CBI with NAFLD group and CBI group

### Factors associated with hepatic steatosis

Two hundred twenty-three children were divided into three groups based on the grading of hepatic steatosis, 58, 51 and 114 of which were mild, moderate and severe steatosis, respectively. There were no statistical differences in hepatic necroinflammation and fibrosis among the three groups. In mild steatosis group, there were 46/58(74.2%) NAFLD with CBI children. While, only 5/114(8%) NAFLD co-existed CBI children were in severe steatosis, (*P* < 0.001) (Table S1) suggesting that NAFLD children co-existed CBI had milder degree of hepatic steatosis than simple NAFLD children. Furthermore, by multivariate analysis, CBI was indeed inversely associated with the degree of hepatic steatosis [odd ratio (OR) 0.037, 95% confidence interval (CI) 0.014–0.098], adjusted for age, gender, body mass index (BMI) z-score, CBI, platelet, alanine aminotransferase (ALT), glutamyl transferases (GGT), albumin, prealbumin, cholinesterase, uric acid, total cholesterol (TC), triglyceride (TG), high density lipoprotein cholesterol (HDL-C), low density lipoprotein cholesterol (LDL-C), the stage of fibrosis and the grade of hepatic necroinflammation. In addition, platelet (OR 1.008, 95%CI 1.003–1.014) and TC (OR 2.479, 95%CI 1.082–5.678) were positively associated with the severity of hepatic steatosis (Table 2).

**Table 2:** Associated factors with hepatic steatosis in 223 NAFLD pediatric individuals (Multivariate Analysis)

Variable	Univariate Analysis			Multivariate Analysis		
	***β***	**OR (95% CI)**	***P*-value**	***β***	**OR (95% CI)**	***P*-value**
CBI (Y/N)	-3.455	0.032(0.015–0.067)	0.000	-3.308	0.037 (0.014–0.098)	0.000
Platelet	0.008	1.008(1.004–1.011)	0.000	0.008	1.008 (1.003–1.014)	0.002
TC	0.557	1.746(1.326–2.299)	0.000	0.921	2.479 (1.082–5.678)	0.032

*Y/N* Yes/No, *TC* Total cholesterol, *CBI* Chronic hepatitis B infection

### Factors associated with hepatic necroinflammation

126/223 children were assessed as mild necroinflammation (A ≤ 1), and 97/223 children were severe necroinflammation (A > 1). There was no significant difference in both the grades of hepatic steatosis and the stage of inflammation between the two groups. However, the prevalence of CBI in children with mild necroinflammation was significantly lower than that individuals with severe necroinflammation (22.2% vs. 35.7%, *P* = 0.035) (Table S2). Furthermore, multivariate analysis showed that CBI was an independent factor associated with the severity of hepatic necroinflammation after adjusting for age, gender, BMI z-score, CBI, globulin, ALT, aspartate aminotransferase (AST), albumin, TC, TG, HDL-C, LDL-C, the stage of fibrosis and the degree of steatosis in NAFLD children (OR 6.125, 95%CI 1.958–19.158). Other independently associated factors of the severity of hepatic necroinflammation were hepatic fibrosis (OR 6.252, 95%CI 3.152–12.298) and globulin (OR 0.843, 95%CI 0.763–0.932 (Table 3).

**Table 3:** Associated factors with hepatic necroinflammation in 223 NAFLD pediatric individuals (Multivariate Analysis)

Variable	Univariate Analysis			Multivariate Analysis		
	***β***	**OR (95% CI)**	***P*-value**	***β***	**OR (95% CI)**	***P*-value**
Hepatic fibrosis (F > 1/F ≤ 1)	1.546	4.693 (2.579–8.539)	0.000	1.737	5.681 (2.602–12.401)	0.000
CBI	0.635	1.886 (1.033–3.446)	0.000	1.812	6.125 (1.958–19.158)	0.002
Globulin	-0.092	0.912 (0.849–0.979)	0.011	-0.170	0.843 (0.763–0.932)	0.001

*CBI* Chronic hepatitis B infection, *ALT* Alanine aminotransferase, *TC* Total cholesterol

### Factors associated with significant hepatic fibrosis

Another striking factor that affects the progression of NAFLD is hepatic fibrosis. In order to find out the culprit associated with hepatic fibrosis, 223 children with NAFLD were divided into no or mild fibrosis group (F ≤ 1) and significant fibrosis group (F > 1). Individuals with significant fibrosis presented more severe hepatic necroinflammation compared to no or mild fibrosis (61.4% vs. 24.8%, *P* = 0.001). And AST, not ALT was higher in F > 1 group than F ≤ 1 group (*P* = 0.001), which was consistent with histologically hepatic necroinflammation. As to hepatic steatosis, there were no significant difference between the two groups (Table S3). Furthermore, in the multivariate analysis, hepatic necroinflammation was still an independent factor associated with the severity of hepatic fibrosis after adjusting for age, gender, BMI z-score, CBI, hemoglobin, Prealbumin, total bilirubin (TBil), alkaline phosphatase (ALP), total bile acid (TBA), creatinine, amylase, urid acid, AST, the grade of necroinflammation and the degree of steatosis in NAFLD children (OR 4.504, 95%CI 2.045–9.920). In addition, creatine was positively associated with the severity of fibrosis (OR 1.008, 95%CI 1.003–1.014) (Table 4). Of note, co-existing CBI was not associated with the severity hepatic fibrosis in NAFLD pediatric population.

**Table 4:** Associated factors with hepatic fibrosis in 223 NAFLD pediatric individuals (Multivariate Analysis)

Variable	Univariate Analysis			Multivariate Analysis		
	***β***	**OR (95% CI)**	***P*-value**	***β***	**OR (95% CI)**	***P*-value**
Grade of Necro. (A > 1/A ≤ 1)	1.575	4.832(2.717–8.592)	0.000	1.501	4.504 (2.045–9.920)	0.000
Creatinine	-0.049	0.952(0.931–0.974)	0.000	0.008	1.008 (1.003–1.014)	0.002

*Grade of Necro.* Grade of necroinflammation

## Discussion

This is a retrospective study, with hepatic biopsy-proven assessment, to explore the effect of CBI on hepatic steatosis, necroinflammation and fibrosis in NAFLD pediatric population. We found that CBI was inversely associated with hepatic steatosis and positively associated with hepatic necroinflammation, however, no clear association were observed between CBI and hepatic fibrosis in NAFLD pediatric population. In addition, there was a positive association between hepatic necroinflammation and hepatic fibrosis in NAFLD children.

Previous studies on correlations between NAFLD and HBV infection reported conflicting results and were mostly based on the diagnosis and assessment methods of imaging or invasive serum marker, ignoring critical information provided by histological features. A large prospective cohort study using ultrasounds showed that HBsAg ( +) as a lower risk predictor that was associated with developing NAFLD in HBsAg ( +) and health population, indicating that HBV play a protective role on the development of hepatic steatosis [9]. In support of our studies, an ultrasonography-aided health checkup cohort of 33439 volunteers also demonstrated an inverse association between HBV infection and hepatic steatosis [10]. The above studies suggested that there was an inextricable association between HBsAg and hepatic steatosis. In addition, HBV DNA appeared to affect hepatic steatosis. A meta-analysis including 17 studies that demonstrated HBV DNA was negatively associated with steatosis [20]. Notably, Liver biopsy is considered as the gold standard for the diagnosis of NAFLD [21], whereas most of previous efforts taking advantages of biopsies have only focused on adult population, leaving childhood NAFLD largely unexplored. Moreover, there was little other studies other than our previous study on the association between HBV and NAFLD in NAFLD population. We have previously shown that CBI was inversely and mutually association with NAFLD, compared to mild steatosis, moderate OR 0.367, 95%CI; severe OR 0.089 between two groups [11]. In this study, we further confirmed that CBI was inversely associated with the grade of steatosis by dividing three groups according to the grade of steatosis in biopsy-proven NAFLD pediatric population. However, only a few studies have investigated the mechanistic cause-effect linkage between NAFLD and CBI. Some studies have reported that individuals with HBV infection have lower cholesterol and triglycerides than general population, possibly affecting liver fatty accumulation [9]. On the other hand, a healthier lifestyle in CBI population could partially explained the result.

Most of the morbidity and mortality associated with chronic hepatic disease results from the development of hepatic necroinflammation and fibrosis. The influence of HCV on the development of NAFLD was well established, however, HBV was not clear. To the best of our knowledge, both CBI and NAFLD were the vital roles in the etiology of hepatic necroinflammation and fibrosis. While, it was paradoxical that NAFLD with coexisting CBI could be either beneficial or detrimental to hepatic disease progression. A study from HBeAg-negative chronic hepatitis B (CHB) patients with hepatic steatosis found that the activity of hepatic inflammation may be associated with NAFLD. Another retrospective study from China study indicated that the presence of steatosis was likely to be positively associated with ALT (OR 2.219, 95% CI 1.223–4.028) [14]. However, mechanisms and causalities were currently unclear. Emerging evidence has demonstrated that hepatic active inflammation may accelerate virus clearance and favored efficient virological response [22]. Interestingly, recent data showed contaminant steatosis in CBI population, no matter in children or in adults, can achieve better viral response that CBI patients with steatosis receiving long‐term antiviral treatment were more likely to have HBsAg loss, which might relate to higher inflammatory activity among CHB co-existed NAFLD. Nevertheless, our findings that CBI was positively associated with hepatic necroinflammation in NAFLD pediatric population. These findings suggested that CBI and NAFLD might synergistically impact on the development of hepatic inflammation. In addition, according to our results, higher stage of fibrosis was associated with hepatic necroinflammation, which are largely in agreement with current literature [23, 24].

CBI and NAFLD have demonstrated distinct effects in the development of hepatic fibrosis and hepatic necroinflammation. A cross-sectional study in 168 CHB with or without steatosis Iran patients showed that viral factors, not steatosis, appears to be associated with both hepatic necroinflammation and fibrosis [25]. Another data from CHB adults in USA also made comparative results [26]. Also, findings from a Greek study reported that advanced fibrosis only associated with age and hepatic necroinflammation, rather than the presence of steatosis [27]. Our data also did not reveal any significant association between co-existed CBI and hepatic fibrosis, even in pediatric NAFLD children.

There are some limitations in our study. Firstly, the direction of the association of CBI with the progression of NAFLD-related liver disease cannot be concluded from a cross-sectional analysis. Secondly, lifestyle related factors that might contribute to steatosis were not included in our analysis. Thirdly, our study mainly focused on HBV genotype B and C, while the other HBV genotype remained to be further explored. In addition, our data from biopsy-proven specimens are rare and precious. Although paired matching could give us more convincing results, it is necessary to find a balance in this study due to the limited sample size. On the other hand, our data from hospitalized pediatric population with liver biopsy may inevitably produce selective population bias, and as a retrospective and observational study, time-lead bias was inevitable. Taken together, future prospective follow-up cohort studies will be set out to explore whether CBI in pediatric NAFLD population affect NAFLD-related hepatic steatosis, necroinflammation and fibrosis. Moreover, future study would focus on lifestyle related factors in CBI population influence fat accumulation from early childhood onwards. For all that, we believe that the valuable data from NAFLD children in this study provided perspectives for understanding the effect of CBI on NAFLD and filled the gap of the association of CBI with NAFLD in the field of NAFLD pediatric population.

## Conclusions

Our study shows that CBI is inverse associated with the grade of steatosis and positively associates with severe hepatic necroinflammation in NAFLD children. However, CBI does not appear to affect significant hepatic fibrosis in NAFLD pediatric population.

### Supplementary Information

**Additional file 1.**

**Additional file 2.**

**Additional file 3.**

## Data Availability

The datasets used and/or analyzed during the current study are available from the corresponding author on reasonable request.

## Abbreviations

NAFLD: Nonalcoholic fatty liver disease
NAFL: Nonalcoholic fatty liver
NASH: Non-alcoholic steatohepatitis
CBI: Chronic hepatitis B infection
CHB: Chronic hepatitis B
MTCT: Mother-to-child transmission
HCC: Hepatocellular carcinoma
BMI: Body mass index
DBil: Direct bilirubin
TBil: Total bilirubin
ALT: Alanine aminotransferase
AST: Aspartate aminotransferase
ALP: Alkaline phosphatase
GGT: Glutamyl transferases
TBA: Total bile acid
TC: Total cholesterol
TG: Triglyceride
HDL-C: High density lipoprotein cholesterol
LDL-C: Low density lipoprotein cholesterol
ApoA1: Apolipoprotein A1
ApoB: Apolipoprotein B
Lp (a): Lipoprotein (a)
PT: Prothrombin time
