# Supplementary material for: Association of chronic hepatitis B infection with hepatic steatosis and injury in nonalcoholic fatty liver disease children
Source: BMC Gastroenterol. 2024 Jan 2;24:2. doi: 10.1186/s12876-023-03103-9 (PMC10759402; doi:10.1186/s12876-023-03103-9)
Supplement: Supplementary file 1 — Additional file 1. [file 12876_2023_3103_MOESM1_ESM.docx]

**Table S1. Characteristics of hepatic steatosis in NAFLD Pediatric Population**

| **Variable** | **Mild**  **(n=58)**  **M(quartile)** | **Moderate**  **(n=51)**  **M(quartile)** | **Severe**  **(n=114)**  **M(quartile)** | ***P*-value*** |
| --- | --- | --- | --- | --- |
| Age (years) | 11 (5-16) | 12 (9-15) | 12 (10-13) | 0.571 |
| Gender (M/F) | 40/8 | 42/9 | 103/11 | 0.334 |
| Platelet (10^^9^/L) | 247.5 (187.3-277.0) | 273.0 (234.0-336.0) | 287.0 (251.8-335.0) | ＜0.001 |
| Albumin (g/L) | 42.0 (40.0-45.3) | 43.0 (41.0-47.0) | 45.0 (43.0-48.0) | 0.001 |
| BMI z-score | -0.02 (-0.62-0.52) | 0.05 (-0.76-0.78) | 0.01 (-0.83-0.63) | 0.879 |
| Prealbumin (mg/L) | 163.0 (139.5-203.3) | 226.0 (190.0-265.0) | 229.5 (203.8-261.3) | ＜0.001 |
| ALT (U/L) | 86.5 (49.8-143.0) | 129.0 (75.0-186.0) | 150.0 (86.8-259.8) | ＜0.001 |
| AST (U/L) | 68.0 (39.8-83.8) | 79.0 (47.0-101.0) | 80.0 (55.3-135.5) | 0.065 |
| GGT (U/L) | 31.0 (21.6-54.3) | 43.0 (26.0-66.0) | 61.0 (44.0-97.0) | ＜0.001 |
| ALP (U/L) | 262.5 (196.3-312.8) | 256.0 (160.0-312.0) | 291.5 (210.0-382.0) | 0.022 |
| Cholinesterase (U/L) | 7367.0 (6710.0-8985.3) | 9080.0 (7525.0-10083.0) | 10080.0 (8814.0-10991.3) | ＜0.001 |
| PT (s) | 11.4 (10.9-12.1) | 11.1 (10.7-11.6) | 10.9 (10.3-11.3) | ＜0.001 |
| TC (mmol/L) | 3.7 (3.3-4.2) | 4.4 (3.9-5.0) | 4.4 (3.8-5.0) | ＜0.001 |
| TG (mmol/L) | 1.0 (0.8-1.5) | 1.3 (0.9-2.0) | 1.5 (1.1-2.0) | ＜0.001 |
| LDL-C (mmol/L) | 2.3 (2.0-2.9) | 3.0 (2.6-3.3) | 3.0 (2.5-3.5) | ＜0.001 |
| ApoB (mmol/L) | 0.6 (0.5-0.7) | 0.8 (0.6-1.0) | 0.8 (0.7-1.0) | ＜0.001 |
| CBI (n, %) | 46 (74.2) | 11 (17.7) | 5 (8) | ＜0.001 |
| **Stage of Fibrosis** |  |  |  | 0.706 |
| S0-1 (n, %) | 26 (44.8%) | 32 (62.7%) | 51 (44.7%) |  |
| S2-4 (n, %) | 32 (55.2%) | 19 (37.35) | 63 (55.35) |  |
| **Grade of Necro.** |  |  |  | 0.706 |
| A0-1 (n, %) | 28 (48.3%) | 36 (70.6%) | 62 (54.4%) |  |
| A2-3 (n, %) | 30 (51.7%) | 15 (29.4) | 52 (45.6%) |  |

“*****” means the *P*-value among the degree of the hepatic steatosis group.

Abbreviation: ALT, alanine aminotransferase; AST, aspartate aminotransferase; GGT, glutamyl transferases; ALP, alkaline phosphatase; PT, prothrombin time; TC , total cholesterol; TG, triglyceride; LDL-C , low density lipoprotein cholesterol; ApoB, apolipoprotein B; CBI, chronic hepatitis B infection; Grade of Necro., Grade of necroinflammation.
