# Supplementary material for: Association of chronic hepatitis B infection with hepatic steatosis and injury in nonalcoholic fatty liver disease children
Source: BMC Gastroenterol. 2024 Jan 2;24:2. doi: 10.1186/s12876-023-03103-9 (PMC10759402; doi:10.1186/s12876-023-03103-9)
Supplement: Supplementary file 2 — Additional file 2. [file 12876_2023_3103_MOESM2_ESM.docx]

**Table S2. Characteristics of Grade of Necroinflammation in NAFLD Pediatric Population**

| **Variable** | **A≤1**  **(n=126)**  **M(quartile)** | **A＞1**  **(n=97)**  **M(quartile)** | ***P*-value*** |
| --- | --- | --- | --- |
| Age(years) | 12 (10-15) | 11 (10-13) | 0.394 |
| Gender (M/F) | 111/15 | 84/13 | 0.739 |
| BMI z-score | 0.08 (-0.83-0.68) | -0.04 (-0.74-0.54) | 0.703 |
| Globulin (g/L) | 26 (24-29) | 27 (25-30) | 0.008 |
| Prealbumin (mg/L) | 230 (197-267) | 197 (143-232) | <0.001 |
| ALP (U/L) | 261 (175-312) | 295 (218-372) | 0.005 |
| TBA (umol/L) | 6 (3-8) | 7 (4-10) | 0.003 |
| Creatinine (umol/L) | 55 (46-68) | 53 (46-57) | 0.022 |
| Cholinesterase (U/L) | 9674.5 (7820.3-10492.0) | 8906.0 (7286.0-10264.5) | 0.066 |
| Urid acid (umol/L) | 382 (285-444) | 344 (272-404) | 0.027 |
| GGT (U/L) | 45 (26-67) | 60 (38-100) | 0.002 |
| ALT (U/L) | 98 (62-164) | 152 (97-293) | <0.001 |
| AST (U/L) | 65 (38-83) | 94 (68-168) | 0.002 |
| PT(s) | 10.9 (10.4-11.4) | 11.3 (10.8-11.9) | <0.001 |
| TC (mmol/L) | 4.23 (3.66-4.98) | 4.18 (3.67-4.62) | 0.542 |
| TG (mmol/L) | 1.38 (0.92-1.95) | 1.28 (0.97-1.64) | 0.381 |
| HDL-C (mmol/L) | 1.10 (0.99-1.32) | 1.11 (1.00-1.29) | 0.957 |
| LDL-C (mmol/L) | 2.84 (2.31-3.28) | 2.88 (2.31-3.22) | 0.895 |
| ApoA1 (mmol/L) | 1.22 (1.11-1.36) | 1.22 (1.13-1.38) | 0.904 |
| ApoB (mmol/L) | 0.78 (0.61-0.92) | 0.78 (0.61-0.93) | 0.945 |
| Lp (a) (mmol/L) | 46.5 (30.8-93.5) | 46.0 (23.0-68.0) | 0.222 |
| CBI (n, %) | 28 (22.2) | 34 (35.7) | 0.036 |
| **Hepatic steatosis** |  |  | 0.706 |
| Mild (n, %) | 28 (22.2%) | 30 (30.9%) |  |
| Moderate (n, %) | 36 (28.6%) | 15 (15.5%) |  |
| Severe (n, %) | 62 (49.2%) | 52 (53.6%) |  |
| **Stage of Fibrosis** |  |  | <0.001 |
| S0-1 (n, %) | 82 (65.1%) | 27 (27.8%) |  |
| S2-4 (n, %) | 44 (34.9%) | 70 (72.2%) |  |

“*****” means the *P*-value between A≤1 group and A＞1 group

Abbreviation: ALP, alkaline phosphatase; BMI, body mass index; TBA, total bile acid; GGT, glutamyl transferases; ALT, alanine aminotransferase; AST, aspartate aminotransferase; PT, prothrombin time; TC , total cholesterol; TG, triglyceride; HDL-C, high density lipoprotein cholesterol; LDL-C , low density lipoprotein cholesterol; ApoA1, apolipoprotein A1; ApoB, apolipoprotein B; Lp (a), lipoprotein (a); CBI, chronic hepatitis B infection.
