# Supplementary material for: Association of chronic hepatitis B infection with hepatic steatosis and injury in nonalcoholic fatty liver disease children
Source: BMC Gastroenterol. 2024 Jan 2;24:2. doi: 10.1186/s12876-023-03103-9 (PMC10759402; doi:10.1186/s12876-023-03103-9)
Supplement: Supplementary file 3 — Additional file 3. [file 12876_2023_3103_MOESM3_ESM.docx]

**Table S3. Characteristics of Stage of Fibrosis in NAFLD Pediatric Population**

| **Variable** | **F≤1**  **(n=109)**  **M(quartile)** | **F＞1**  **(n=114)**  **M(quartile)** | ***P*-value*** |
| --- | --- | --- | --- |
| Age(years) | 12 (10-16) | 11 (8-13) | 0.008 |
| Gender (M/F) | 98/11 | 97/17 | 0.279 |
| BMI z-score | 0.12 (-0.48-0.69) | -0.10 (-1.05-0.48) | 0.051 |
| Hemoglobin (g/L) | 138 (131-151) | 135 (128-141) | 0.001 |
| Prealbumin (mg/L) | 232 (199-269) | 210 (154-237) | <0.001 |
| DBil (umol/L) | 3.4 (2.7-4.7) | 2.9 (2.2-4.1) | 0.008 |
| TBil (umol/L) | 9.2 (7.2-12.7) | 7.7 (6.3-10.4) | 0.008 |
| ALP (U/L) | 255 (163-314) | 287 (218-365) | 0.002 |
| TBA (umol/L) | 6 (3-8) | 7 (4-10) | 0.008 |
| Creatinine (umol/L) | 56 (49-69) | 51 (45-57) | <0.001 |
| Amylase (U/L) | 52 (42-63) | 44 (35-57) | 0.003 |
| Urid acid (umol/L) | 382 (317-442) | 346 (246-410) | 0.001 |
| ALT (U/L) | 110 (77-171) | 129 (74-265) | 0.252 |
| AST (U/L) | 72 (42-94) | 80 (59-141) | 0.001 |
| TC (mmol/L) | 4.15 (3.66-4.74) | 4.29 (3.66-4.83) | 0.494 |
| TG (mmol/L) | 1.26 (0.89-1.72) | 1.39 (0.96-1.84) | 0.176 |
| HDL-C (mmol/L) | 1.11 (1.02-1.32) | 1.10 (0.95-1.29) | 0.188 |
| LDL-C (mmol/L) | 2.77 (2.31-3.19) | 2.94 (2.31-3.29) | 0.516 |
| ApoA1 (mmol/L) | 1.22 (1.13-1.37) | 1.22 (1.12-1.37) | 0.757 |
| ApoB (mmol/L) | 0.74 (0.61-0.92) | 0.78 (0.61-0.95) | 0.580 |
| Lp (a) (mmol/L) | 47 (30-86) | 46 (28-74) | 0.660 |
| CBI (n, %) | 30 (27.5) | 32 (28.1) | 1.000 |
| **Hepatic steatosis** |  |  | 0.142 |
| Mild (n, %) | 26 (23.9%) | 32 (28.1%) |  |
| Moderate (n, %) | 32 (29.4%) | 19 (16.7%) |  |
| Severe (n, %) | 51 (46.8%) | 63 (55.3%) |  |
| **Grade of Necro.** |  |  | <0.001 |
| A0-1 (n, %) | 82 (75.2%) | 44 (38.6%) |  |
| A2-3 (n, %) | 27 (24.8%) | 70 (61.4%) |  |

“*****” means the *P*-value between F≤1 group and F＞1 group.

Abbreviation: DBil, direct bilirubin; TBil, total bilirubin; ALP, alkaline phosphatase; TBA, total bile acid; ALT, alanine aminotransferase; AST, aspartate aminotransferase; TC, total cholesterol; TG, triglyceride; HDL-C, high-density lipoprotein cholesterol; LDL-C, low-density lipoprotein cholesterol; ApoA1, apolipoprotein A1; ApoB, apolipoprotein B; Lp (a), lipoprotein (a); CBI, chronic hepatitis B infection.
